# Robot-mediated overground gait training for transfemoral amputees with a powered bilateral hip orthosis: a pilot study

**DOI:** 10.1186/s12984-021-00902-7

**Published:** 2021-07-03

**Authors:** Clara Beatriz Sanz-Morère, Elena Martini, Barbara Meoni, Gabriele Arnetoli, Antonella Giffone, Stefano Doronzio, Chiara Fanciullacci, Andrea Parri, Roberto Conti, Francesco Giovacchini, Þór Friðriksson, Duane Romo, Simona Crea, Raffaele Molino-Lova, Nicola Vitiello

**Affiliations:** 1 The BioRobotics Institute, Scuola Superiore Sant’Anna, 56025 Pontedera, Pisa Italy; 2 IRCCS Fondazione Don Carlo Gnocchi ONLUS, 50143 Florence, Italy; 3 IUVO S.R.L, Via Puglie, 9, 56025 Pontedera, Pisa Italy; 4 Össur, Grjótháls 5, 110 Reykjavík, Iceland; 5 Department of Excellence in Robotics and AI, Scuola Superiore Sant’Anna, 56127 Pisa, Italy

**Keywords:** Transfemoral amputees, Gait training, Gait rehabilitation, Hip orthosis, Exoskeleton, Overground walking

## Abstract

**Background:**

Transfemoral amputation is a serious intervention that alters the locomotion pattern, leading to secondary disorders and reduced quality of life. The outcomes of current gait rehabilitation for TFAs seem to be highly dependent on factors such as the duration and intensity of the treatment and the age or etiology of the patient. Although the use of robotic assistance for prosthetic gait rehabilitation has been limited, robotic technologies have demonstrated positive rehabilitative effects for other mobility disorders and may thus offer a promising solution for the restoration of healthy gait in TFAs. This study therefore explored the feasibility of using a bilateral powered hip orthosis (APO) to train the gait of community-ambulating TFAs and the effects on their walking abilities.

**Methods:**

Seven participants (46–71 years old with different mobility levels) were included in the study and assigned to one of two groups (namely *Symmetry* and *Speed* groups) according to their prosthesis type, mobility level, and prior experience with the exoskeleton. Each participant engaged in a maximum of 12 sessions, divided into one *Enrollment* session, one *Tuning* session, two *Assessment* sessions (conducted before and after the training program), and eight *Training* sessions, each consisting of 20 minutes of robotically assisted overground walking combined with additional tasks. The two groups were assisted by different torque-phase profiles, aiming at improving symmetry for the *Symmetry* group and at maximizing the net power transferred by the APO for the *Speed* group. During the *Assessment* sessions, participants performed two 6-min walking tests (6mWTs), one with (*Exo*) and one without (*NoExo*) the exoskeleton, at either maximal (*Symmetry* group) or self-selected (*Speed* group) speed. Spatio-temporal gait parameters were recorded by commercial measurement equipment as well as by the APO sensors, and metabolic efficiency was estimated via the Cost of Transport (CoT). Additionally, kinetic and kinematic data were recorded before and after treatment in the *NoExo* condition.

**Results:**

The one-month training protocol was found to be a feasible strategy to train TFAs, as all participants smoothly completed the clinical protocol with no relevant mechanical failures of the APO. The walking performance of participants improved after the training. During the 6mWT in *NoExo*, participants in the *Symmetry* and *Speed* groups respectively walked 17.4% and 11.7% farther and increased walking speed by 13.7% and 17.9%, with improved temporal and spatial symmetry for the former group and decreased energetic expenditure for the latter. Gait analysis showed that ankle power, step width, and hip kinematics were modified towards healthy reference levels in both groups. In the *Exo* condition metabolic efficiency was reduced by 3% for the *Symmetry* group and more than 20% for the *Speed* group.

**Conclusions:**

This study presents the first pilot study to apply a wearable robotic orthosis (APO) to assist TFAs in an overground gait rehabilitation program. The proposed APO-assisted training program was demonstrated as a feasible strategy to train TFAs in a rehabilitation setting. Subjects improved their walking abilities, although further studies are required to evaluate the effectiveness of the APO compared to other gait interventions. Future protocols will include a lighter version of the APO along with optimized assistive strategies.

## Background

Transfemoral amputation results primarily from traumatic events, cancer, or vascular disease, the latter accounting for over 80% of cases and most commonly associated with diabetes [1]. Due to population aging, the number of people undergoing amputations is predicted to double by 2050 [1–3]. Moreover, average medical costs for each transfemoral amputee (TFA) exceed $55,000, more than half of which is spent after initial hospitalization, related to outpatient or home treatment [3].

Apart from healthcare expenses, transfemoral amputation has serious repercussions on the overall health and quality of life, largely because of its consequences on locomotion. Community-ambulating TFAs exhibit altered locomotion patterns in activities of daily living (ADLs) and thus rarely achieve functional outcomes comparable to pre-amputation. Indeed, during walking, TFAs employ compensatory strategies mostly due to the decreased capacity for push-off on the prosthetic side. For instance, TFA gait is highly asymmetric, with a shorter stance phase and longer step length on the prosthetic side. Also, hip moments of the sound limb are increased by 17% in flexion and 60% in extension, which results in abnormal force exertion by muscles [4, 5]. Consequently, TFAs present highly energetically-inefficient locomotion and consume approximately 65% more energy than able-bodied individuals, while walking at half the speed [6, 7]. Due in part to gait asymmetry, the prosthetic leg is insufficiently loaded, with a higher risk of osteopenia and osteoporosis of the stump, while the sound limb is overloaded, with a consequent risk elevation for osteoarthritis. In addition, asymmetrical gait increases the incidence of low-back pain [8]. Similarly, stair negotiation is generally performed in a step-by-step manner which requires higher knee extensor moments and power on the sound side, while sit-to-stand transitions are achieved by loading almost all the weight on the sound leg [9, 10]. For all these reasons, TFAs are generally required to adjust their lifestyle by changing jobs or modifying their leisure activities, which can drastically reduce their quality of life and participation in society [11–14].

These secondary disorders and consequences may be mitigated by dedicated rehabilitation programs that aim to improve TFAs’ movement abilities. In the state of the art, outcomes of post-amputation gait rehabilitation of TFAs seem to be highly dependent on factors such as the duration and intensity of the treatment and the age or etiology of the patient. First, TFAs who benefit from a longer rehabilitation treatment demonstrate higher mobility potential compared to patients involved in shorter care [15]. Similarly, functional recovery is fostered by physical therapy delivered in comprehensive inpatient rehabilitation units [16]. Interestingly, the delay between amputation discharge and the start of rehabilitation does not seem to be critical to obtain a comparable level of functional recovery, as measured with the Functional Independence Measure [17]. Moreover, the literature reports different findings on the proportion of TFAs becoming functional prosthetic ambulators after rehabilitation. Some studies found that 62% of TFAs were able to perform the majority of outdoor activities, including the most complex ones such as stair negotiation, whereas others reported that only 25% of TFAs reach the level of community ambulation [18, 19]. This variability might be related to the respective populations involved in each study, as dysvascular amputees have shown lower ambulatory potential than non-dysvascular cases [19, 20].

Different rehabilitative methods have been proposed and investigated for training TFAs. Many programs include supervised overground or treadmill-based gait training, which have been effective in improving spatiotemporal gait parameters of TFAs [21, 22]. Diverse walking modalities have also been explored, for instance by providing TFAs with biofeedback, weight-support, virtual reality (VR), or treadmill walking [23–27]. Other studies have used different rehabilitation approaches, combining psychological and physiotherapeutic treatments, including diverse ADLs [28, 29], or substituting the current prosthesis with a more advanced one [30, 31].

In the last decade, the use of robots –both cartesian manipulators and exoskeletons– has shown promising results in the rehabilitation of motor deficits thanks to the implementation of assist-as-needed paradigms, mostly in the functional recovery of neurological patients [32, 33]. Literature has also supported the idea that robot-assisted rehabilitation could bring several advantages over traditional techniques [34–36]. For instance, powered exoskeletons can improve treatment repeatability by delivering nearly identical torque profiles throughout the rehabilitation process, which cannot be achieved by manual assistance or verbal feedback from the physiotherapist. Moreover, the device power provided can be tuned to target specific gait abnormalities in a controlled way, by implementing subject-dependent assist-as-needed paradigms. A recent trend in rehabilitation robotics has been the use of extremely light-weight single-joint exoskeletons for gait training [37], which, in contrast to treadmill-based gait assistive robots, enable training in everyday scenarios, such as in overground walking and stair negotiation. Among them, powered hip orthoses analogous to our exoskeleton seem to positively support the rehabilitation of stroke survivors and other fragile patients with limited movement abilities, by improving spatiotemporal gait symmetry, metabolic efficiency and kinematics quality [38–41]. Considering that TFAs overload the sound limb and adopt altered biomechanical patterns to compensate for missing power on the prosthetic side, a portable hip orthosis able to deliver positive power in the critical phases of walking—such as the prosthetic push-off phase—may be a promising solution to restore healthier gait and reduce the excessive fatigue of TFAs during walking.

Here, we explored the use of a bilateral powered hip orthosis—the Active Pelvis Orthosis (APO)—for the rehabilitation of TFAs. The main objectives of this pilot study were to verify the feasibility of a one-month robotic APO-mediated training program for TFAs and to investigate exoskeleton training effects on two main gait parameters: symmetry and speed. As secondary outcomes, the study monitored other relevant gait parameters such as metabolic cost, step width, and gait kinematics. Finally, the study also investigated whether those gait parameters were modified when walking with the APO compared to walking without it. This study thus provides the first insight into the response of TFAs to a mobility-based overground training program aided by a robotic hip exoskeleton, and the results are expected to be useful for the design of future rehabilitation methods and studies for TFAs.

## Methods

### Study participants

The study participants were recruited among individuals with unilateral transfemoral amputation, aged between 30 and 80 years old, who had completed the post-amputation rehabilitation course and had a residual mobility level equal to or lower than K3 (Medicare Functional Classification Levels [42]). Exclusion criteria included: (1) relevant comorbidities (e.g., hemiplegia, degenerative nervous system diseases, hip or knee replacement, chronic heart failure, chronic obstructive pulmonary disease, severe sensory deficits, etc.); (2) stump pain or issues with socket fitting; (3) inability to walk on a treadmill; (4) poor cognitive skills (Mini-Mental State Examination < 24 [43]); (5) severe anxiety or depression (State-Trait Anxiety Inventory-Y > 60 [44] and Beck Depression Inventory-II > 29 [45], respectively); (6) implanted cardiac devices, such as pacemakers or automatic defibrillators. Seven participants were enrolled for the study, aged between 46 and 71 years old. All participants provided written informed consent before the first experimental session. Considering its exploratory purpose, the study included participants with diverse anthropometries, prosthesis type, mobility levels (based on their Medicare Functional Classification Level), and prior experience using the APO. In order to provide personalized mobility-based training, participants with higher mobility and/or previous experience with the APO were trained with assistive APO torque profiles that aimed at improving gait symmetry (*Symmetry* group, composed of ID1, ID2 and ID3) whereas those with lower mobility, a mechanical prosthesis and/or no experience using the APO received an assistive action intended to increase their walking speed (*Speed* group, composed of ID4, ID5, ID6 and ID7). Additional participant details can be found in Table 1.

**Table 1:** General data of the participants of the clinical study

		ID1	ID2	ID3	ID4	ID5	ID6	ID7
Personal data	Age	71	61	52	46	62	56	61
	Sex	M	M	M	M	M	F	M
	Height [m]	1.80	1.78	1.66	1.62	1.61	1.69	1.77
	Weight [kg]	70	93	73	98	100	78	91
	Medicare Functional Level	K3	K2	K3	K2	K2	K2	K3
	Experience using APO	Yes	Yes	Yes	No	No	No	No
Amputation	Side	Right	Left	Left	Right	Right	Right	Right
	Additional assistive devices	–	–	–	–	–	Crutches	–
	Level	Proximal	Mid-thigh	Distal	Distal	Mid-thigh	Distal	Distal
	Cause	Trauma	Vascular	Trauma	Vascular	Trauma	Neoplasm	Infection
	Year	2003	2015	1981	2017	1979	2016	2017
Prosthesis	Knee	*Electr*	*Mech*	*Mech*	*Mech*	*Mech*	*Mech*	*Mech*
	Foot	ESAR	ESAR	ESAR	Rigid	Rigid	Rigid	Rigid
Protocol	Group	*Symmetry*	*Symmetry*	*Symmetry*	*Speed*	*Speed*	*Speed*	*Speed*
	# Training sessions	8	8	8	8	5	8	5
	Outcome	Completed	Completed	Completed	Completed	Completed	Completed	Completed

*ESAR* energy storing and return, *Mech* mechanical knee joint, *Electr* electronic knee joint

### The active pelvis orthosis

The Active Pelvis Orthosis (APO) is a bilateral powered robotic exoskeleton designed to gently power hip movements by providing smooth assistive torque at the pelvis level, adapting to natural gait variations. The APO can provide support to individuals with mild gait impairments, i.e. individuals with stepping abilities who could benefit from an assistive hip flexion–extension torque.

The APO system used for this study was based on the same mechatronic architecture as previously-reported prototypes [41, 46], with additional design optimizations for portability and weight reduction to 6.5 kg.

The APO is built around a carbon fiber frame, which surrounds the user’s hips and posterior pelvis, and interfaces with the trunk via a custom spine brace prototyped by Össur (Reykjavik, Iceland). The APO frame carries a backpack–housing the control electronics and battery pack–and two actuation units, one on each side, employing a series elastic actuator architecture [47]. Each actuation unit is deployed along two parallel axes. The first axis (posteriorly located) is the output shaft of the DC motor coupled with a 1:100 reduction stage and a torsional spring. The custom-designed APO torsional spring ensures a compliant interaction with the lower-limb segment, with a stiffness of 220 N∙m/rad, and can deliver a maximal torque of 36 N∙m. The torsional spring deformation is measured using a 17-bit absolute encoder and used to compute the actual output torque in real-time. The second axis (anteriorly located) is collocated with the hip flexion–extension axis and is equipped with a 13-bit absolute encoder for hip angle measurement. The two parallel axes are connected by a 4-bar linkage mechanism. For each side, the transfer of assistive torque from the actuation unit to the hip articulation is guaranteed by a thigh cuff connected to the actuation output axis through a rigid link. Each actuation unit can deliver a peak torque of 17 N·m over a range of movement (RoM) of [− 20–100] deg and features a passive degree of freedom allowing the user to freely perform hip ab/adduction movements in the RoM [− 15–30] deg.

The APO can deliver the desired torque pattern through a hierarchical control algorithm relying on accurate gait phase recognition for synchronization of the assistive action with the intended movement of the user. The APO control system runs on a real-time controller (NI SbRIO9651 processor, National Instruments, Austin, Texas, US) featuring both a dual-core ARM controller and a Field-Programmable Gate Array (FPGA) processor. A proportional-integral closed-loop torque compensator running on the FPGA at 1 kHz is responsible for tracking the desired assistive torque (i.e., reference torque) to be delivered to the user. A high-level control layer (running at 100 Hz on the ARM processor) calculates the desired torque profile following a precise gait-phase estimation. The gait phase is obtained by continuously tracking the hip kinematics through the embedded joint angle sensors, according to the algorithm presented in [48]. For each leg, a pool of adaptive oscillators is used to estimate a continuous phase variable from the hip angular profile. The phase is smoothly reset at the maximum hip flexion angle of each stride via a phase error compensator that avoids discontinuities.

The relatively low output impedance of the APO combined with the reliable real-time estimate of the gait phase allows the APO to be controlled in two different operational modes, namely *Transparent mode* (TM) and *Assistive mode* (AM). In TM, the motors are enabled and accommodate the movement of the user to maintain at the hip joint an interaction torque equal to 0. The APO is thus *transparent* to the user’s movement and provides minimal-to-null resistance to the user. Indeed, a previous version of the APO with an analogous actuation concept was shown to have only 1 N·m/rad residual output impedance at 1 Hz [46]. In AM, the APO commands the desired torque-phase profile. In this case, the torque profile of each hip joint is computed as the sum of two Gaussian functions assisting both hip flexion and extension [41]. For each Gaussian function, the experimenter can tune the following parameters: the phase and amplitude of the peak torque (respectively in [% of the gait phase] and [N·m]) and the duration of the assistance (i.e., the width of the Gaussian function in [% of the gait phase]).

### Clinical protocol

**Fig. 1:**
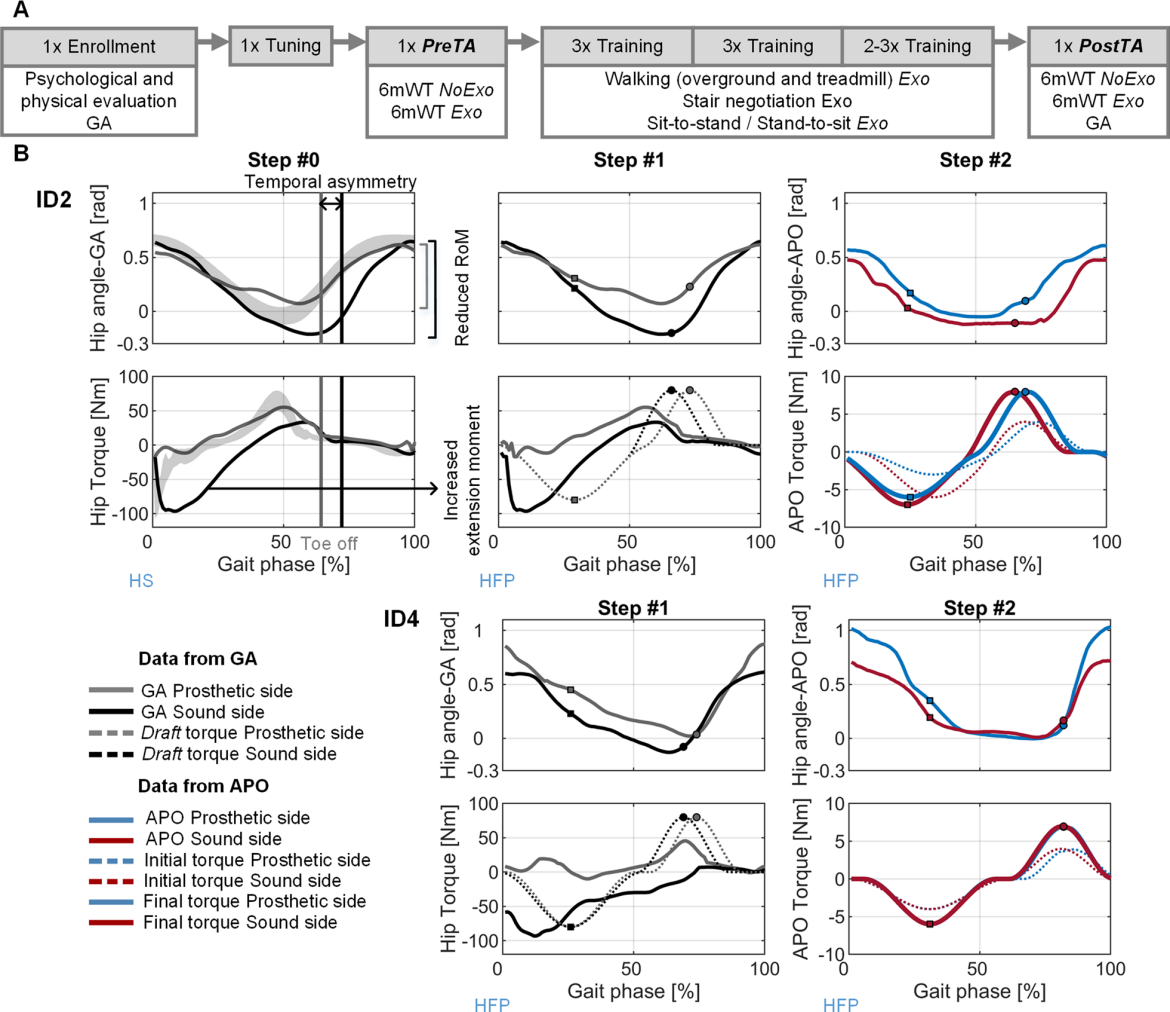
**A.** Experimental protocol: the *Enrollment* session including initial evaluations and GA without the APO (*NoExo*) was followed by a *Tuning* session to select the assistive parameters. Participants then performed a *Pre-training Assessment* session (*PreTA*) with two 6mWTs in *NoExo* and with the APO (*Exo*) and eight *Training* sessions in *Exo*. Last, the *Post-training Assessment* (*PostTA*) was identical to the *PreTA* with a final GA. **B.** Tuning procedure for two representative participants of the *Symmetry* (ID2) and *Speed* (ID4) groups. For the *Symmetry* group, *Step #0* aimed at identifying hip angle abnormalities by comparing hip kinematics with the physiological range (grey area). Then, in *Step #1*, data from the GA—where the 0% of the gait phase corresponded to heel strike (HS)– were shifted to reset the phase at the hip flexion peak (HFP). *Draft* assistive torque profiles were designed (dashed lines) to improve temporal symmetry: on the sound side, the flexion peak (black circle) was delivered earlier than the physiological one to promote an earlier flexion and reduce stance time; on the prosthetic side, the extension peak (grey square) was delayed with respect to the physiological pattern to promote a longer stance. During *Step #2*, each participant walked in TM and AM with the *initial* torque profiles (colored dashed lines); amplitude was gradually increased based on the participant’s preference and phase and duration were fine-tuned. At the end of the session, the final torque profiles (solid bold colored lines) were locked. For the *Speed* group, the *Tuning* procedure started from *Step #1* and the *draft* torque profiles were designed to maximize the net power transferred to the participant: peak torque phases were synchronous to flexion/extension velocity peaks and torque durations corresponded to flexion/extension durations. *Step #2* and *Step #3* followed the same procedure as for the *Symmetry* group

The study was carried out at the clinical center IRCCS Fondazione Don Carlo Gnocchi of Florence (Italy) and lasted approximately ten months. The experimental protocol was approved by the local Ethics Committee, namely the Comitato Area Vasta Centro Toscana (Protocol ID: CLs + + 1stCS; approval number: 12739_spe; ClinicalTrials.gov ID: NCT03296904). This study was classified as an uncontrolled interventional longitudinal case series. For each participant, the protocol included 12 sessions conducted in 12 different days over 4 weeks (Fig. 1A).

In the first (*Enrollment*) session the experimenters verified that each recruited patient complied with inclusion/exclusion criteria. In addition, participants’ kinematics were recorded in a gait analysis (GA) room equipped with an optoelectronics motion capture system (Smart-DX, BTS Bioengineering, Quincy, MA, USA) and force platforms (P6000, BTS Bioengineering, Italy), following the DAVIS protocol [49].

The second (*Tuning*) session was devoted to tuning the assistive profile (torque-phase profile), by tailoring it to the specific gait kinematics of each participant. The strategy adopted for the selection and tuning of the torque profile is detailed hereafter. A different tuning procedure was employed for the *Symmetry* and *Speed* groups. For the *Symmetry* group, the tuning of the assistive profile aimed at supporting the amputees in recovering a more physiological gait pattern. Additional details of the tuning procedure for a representative participant (ID2) can be found in Fig. 1B.

- *Step #0—Identification of relevant abnormalities in hip kinematics:* clinicians analyzed the GA recorded during *Enrollment* with attention to indicators of gait asymmetry in hip kinematics and stance phase duration such as (1) a prolonged hip extension phase on the sound limb and/or (2) a shorter hip extension phase (with correspondingly smaller RoM) on the prosthetic side.
- *Step #1—Draft assistive torque-phase profile:* experimenters made the initial choice of the parameters of the two Gaussian functions composing the torque-phase profile of each hip joint with the goal of reducing the asymmetric gait pattern. Specifically, to decide how to set the phase, amplitude, and duration of each Gaussian function, experimenters compared participants’ hip kinematics with able-bodied normative data with the goal of (1) shortening the sound-limb hip extension by promoting an earlier hip flexion (by delivering a flexion torque at the end of the stance phase) and (2) obtaining a longer hip extension phase for the prosthetic leg (by delivering an extension torque at the end of the swing phase).
- *Step #2—Fine-tuning of the torque-gait-phase profile:* participants were asked to walk in AM with the APO delivering the torque profiles defined in the previous step. Minor refinements to the Gaussian parameters were introduced by analyzing the hip angles tracked by the APO joint encoders in real-time. Torque amplitude was gradually increased with the objective of reaching a peak value of approximately 0.1 N‧m/kg; the final choice for the torque profile was reached by taking into account the participants’ feedback to ensure a comfortable interaction. At the end of *Step #2*, the final assistive torque–phase profile was selected.
- *Step #3—Familiarization:* the final assistive torque profile achieved in *Step #2* resulted in 5–15% phase variations with respect to the initial one from *Step #1*. Such variations are sufficient to result in different outcomes with respect to gait parameters and comfort perception [50, 51]. Thus, in *Step #3* the participant was allowed to try the assistive torque defined in *Step #2* for about 10 min. They were asked to walk back and forth in a corridor of approximately 20 m while concentrating on the assistance, without receiving external inputs until they judged the familiarization time to be sufficient. Participant safety was ensured using an overhead body weight support system comprising a safety harness secured to a sliding track on the ceiling.

For the *Speed* group, the torque-phase profile aimed at maximizing the net power transferred by the APO to the participant. The tuning procedure started from *Step #1* and, for both flexion and extension, the draft assistive torque-phase profile was tuned to synchronize the Gaussian flexion/extension peaks with the corresponding hip joint velocity peaks provided by the GA. From *Step #2*, the procedure was similar to the one carried out for the *Symmetry* group. Finally, the experimenters selected whether to assist the participant with flexion–extension assistance (as for ID4 and ID6) or with a torque profile assisting only flexion (as for ID5 and ID7). More details are shown in Fig. 1B. The total duration of the *Tuning* procedure and the phase difference between *Step #1* and *Step #2* were indicated as median (min, max).

Following the *Enrollment* and *Tuning* sessions, the participant started the interventional part of the protocol, which consisted of one *Pre-training Assessment (PreTA)*, eight *Training,* and one *Post-training Assessment* (*PostTA*) sessions. In the *PreTA* and *PostTA* sessions, participant’s gait ability was assessed in two conditions, i.e., with (*Exo*) and without (*NoExo*) wearing the exoskeleton. In each session, the participant executed two 6-min Walking Tests (6mWT) [52], respectively in *NoExo* and *Exo* conditions (*NoExo* condition was always executed first). For the 6mWT the participant was asked to walk back and forth a straight 18-/24-m corridor. In the *Exo* condition, in order to ensure a smooth human–robot interaction, the APO assistance was switched off (i.e., APO was operated in TM) when the participants turned around at the end of each corridor: the APO was operated in AM only when participants walked on the straight portion of the corridor. Participants of the *Symmetry* group were instructed to execute all 6mWTs at their maximal walking speed while those in the *Speed* group were instructed to walk at a comfortable speed. In the *PostTA* session, GA was carried out following the same methodology as the *Enrollment* session (without wearing the exoskeleton).

Each participant performed eight *Training* sessions, with a maximum of three sessions per week over a period of three weeks. Each *Training* session consisted of a 20-min assisted overground walking exercise at self-selected speed, in the same corridor where *PreTA* and *PostTA* recordings took place. The subject was allowed to rest at any time during the session, and two physical therapists monitored the hear rate (HR) of the participant when resting for safety purposes. Based on the HR, participants were instructed to increase/decrease walking velocity to ensure a low-to-medium-intensity activity [53, 54]. Even though the main task of these *Training* sessions was overground walking, subjects were also asked to perform treadmill walking and around five minutes of stair negotiation and sit-to-stand/stand-to-sit transitions, assisted as in [55]. The effect of the APO during these additional tasks is beyond the scope of this study and will be reported in future publications.

### Outcome measures and data analysis

All collected data were recorded by the onboard APO sensors and commercial measurement equipment (Smart-DX, OptoGait, Oxycon, Witty) and analyzed in Matlab (MathWorks, Inc., Natick, MA, USA).

Data from the onboard APO sensory system included the hip angular and torque profiles and were collected only in the *Exo* condition. From the hip angle, we estimated the joint velocity and the actual delivered assistive power, the latter computed as the product of the output torque and hip joint velocity. Hip angle, torque, and power signals were then segmented into single strides based on the estimated gait phase, which considered the instant of maximum hip flexion angle as the start (0%) of the stride [48]. Finally, all strides were interpolated and averaged.

During the 6mWTs and the GA performed at the *Assessment* (*PreTA* and *PostTA*) and *Enrollment* sessions, the following data were collected. The overall distance walked was manually calculated based on the length of the corridor and the number of “laps” walked. The average walking speed was measured by means of two photocells (Witty, Microgate S.r.l., Italy) placed 6 m from each other, in the middle of the corridor. In the 6 m between the photocells, an optical detection system (Optogait, Microgate S.r.l., Italy) recorded the cadence, stride, and step length. Optogait data were further analysed to estimate the temporal and spatial Symmetry Indices (SI) for every step, with 0% indicating full symmetry [56]. Non-parametric inference was chosen since normality was not proven for any of the data distributions (Lilliefors test, alpha = 0.05) thus results are presented as the median, maximum and minimum values and the interquartile range (IQR) of all the aforementioned gait parameters during each 6mWT. At the end of each 6mWT, participants were requested to assess the perceived effort through the CR-10 Borg scale [57].

A portable gas analyser (Oxycon Mobile, CareFusion, Germany) was used to estimate the energy expenditure of walking through indirect calorimetry. The system monitored the rates of Oxygen uptake $\left( {VO_{2}^{ \cdot } \left[ {\frac{{ml}}{{\min }}} \right]} \right)$ and Carbon Dioxide output $\left( {V\dot{C}O_{2} \left[ {\frac{{ml}}{{min}}} \right]} \right)$ that are commonly used to compute the energy cost of transport (CoT) according to the Brockway equation [58]. However, since the Respiratory Exchange Ratio (RER) at steady state was higher than at the baseline due to the buffering action of bicarbonates, the use of the Brockway equation could result in an overestimate of the CoT [59]. Thus, in this case, the computation of the CoT was based on the $VO_{2}^{ \cdot }$ instead of the Brockway equation. Baseline consumption was obtained by averaging the values of the last 3 min of a 5-min rest period (patient was sitting during rest) prior to each 6mWT. The baseline mean value was then subtracted from the average steady-state consumption of the last 3 min of each 6mWT to obtain the net oxygen consumption $\left( {VO_{{2net}}^{ \cdot } \left[ {\frac{{ml}}{{min}}} \right]} \right)$. The CoT was computed by normalizing $VO_{{2\,net}}^{ \cdot }$ by the participant’s body mass (BM, kg) and the average speed during the test $\left( {v = \frac{{distance}}{6}~\left[ {\frac{m}{{min}}} \right]} \right)$:

$$CoT = \frac{{VO_{{2\,net}}^{ \cdot } }}{{v \cdot BM}}\left[ {\frac{{ml}}{{kg \cdot m}}} \right].$$

In addition, a pulse oximeter integrated in the system was used to monitor the HR, in order to assess exercise intensity using the percentage of maximal heart rate (HR_max_) as a reference, considering HR_max_ = 220 − age [bpm] and HR_max_ = 200 − age [bpm] for male and female participants, respectively [60].

For each 6mWT, given the limited sample size, the group results of the *Symmetry* and *Speed* groups were obtained by calculating the median, maximum and minimum values, as well as the percentage variations between the median values across all tests, to determine the *Exo* vs*. NoExo* and *PreTA* vs*. PostTA* differences; no comparative statistical analysis (hypothesis testing) was performed. The inter-group differences are shown as median (min, max).

GA outcomes include spatiotemporal parameters as well as full lower-limb kinematics and dynamics, shown in comparison to physiological ranges and profiles, previously obtained by averaging hip angles across a pool of able-bodied individuals. The data from the Smart-DX system were used to compute the hip RoM as the difference between the maximum flexion and extension values and all data were inspected by a physiatrist to identify clinically relevant *PreTA* vs*. PostTA* changes in gait parameters.

Finally, the number of device-related adverse events and failures were manually collected at the end of every session.

## Results

The clinical protocol was safely and smoothly completed by all participants (Table 1) with no reported discomfort deriving from the use of the APO device and no protocol-related adverse events were reported. During the tuning procedure, subjects walked with the exoskeleton for a median time of 39.3 (25.7, 89.9) minutes, of which 25.7 (20.3, 58.7) minutes was with active assistance. The median phase difference between *Step #1* and *Step #2* was 7% (3, 13) in flexion and 5% (0, 10) in extension.

**Fig. 2:**
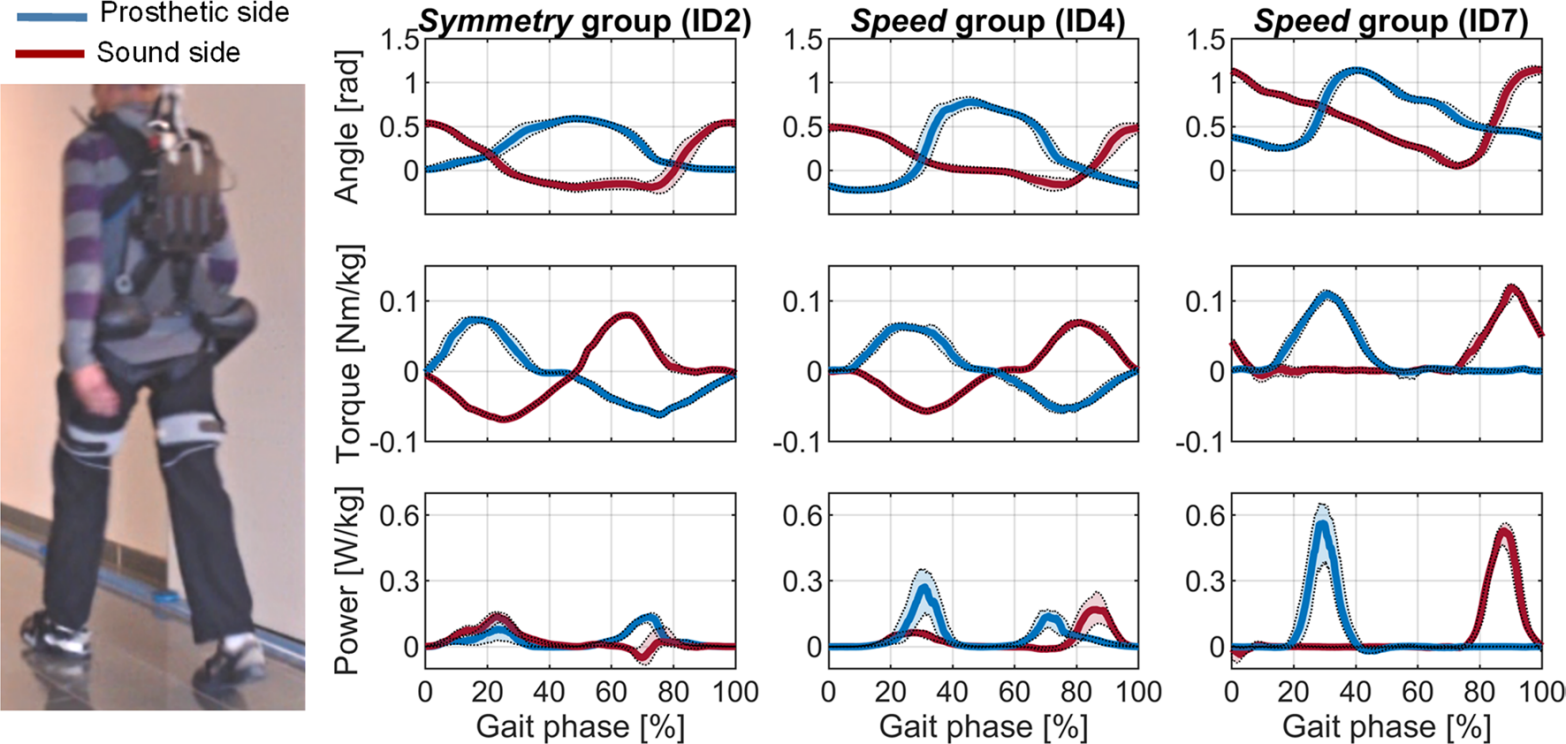
Picture of one of the participants who completed the clinical protocol and example of hip angular profiles, APO output torque and power measured by the onboard sensors for three representative participants during the 6mWT of the *PostTA* session (ID2, ID4 and ID7). Data are shown as median (IQR) for the Sound and Prosthetic sides

Throughout the full study, the APO delivered assistance in 73 total sessions with a non-continuous mean usage of 33 min per session and a total working time of more than 41 h. Participants ID5 and ID7 were trained for just 5 sessions instead of 8 due to their limited personal availability, while all others completed the full 8 sessions. During the 40-h total APO working time, only one minor hardware failure was identified, namely the breakage of one of the plastic shells of the spine brace. The damage of the brace was noted during the protocol of ID5 and ID7, when the experimenters observed that the human–robot coupling was not as stable as expected (i.e., minimum-to-null relative movement between the human trunk and the spine brace). The spine brace was quickly replaced, and the protocol was restarted for ID5 and ID7. Figure 2 depicts representative assistive profiles for three participants (ID2, ID4 and ID7), recorded during the 6mWTs in the *Exo* condition of the *PostTA.*

### 6mWT

**Fig. 3:**
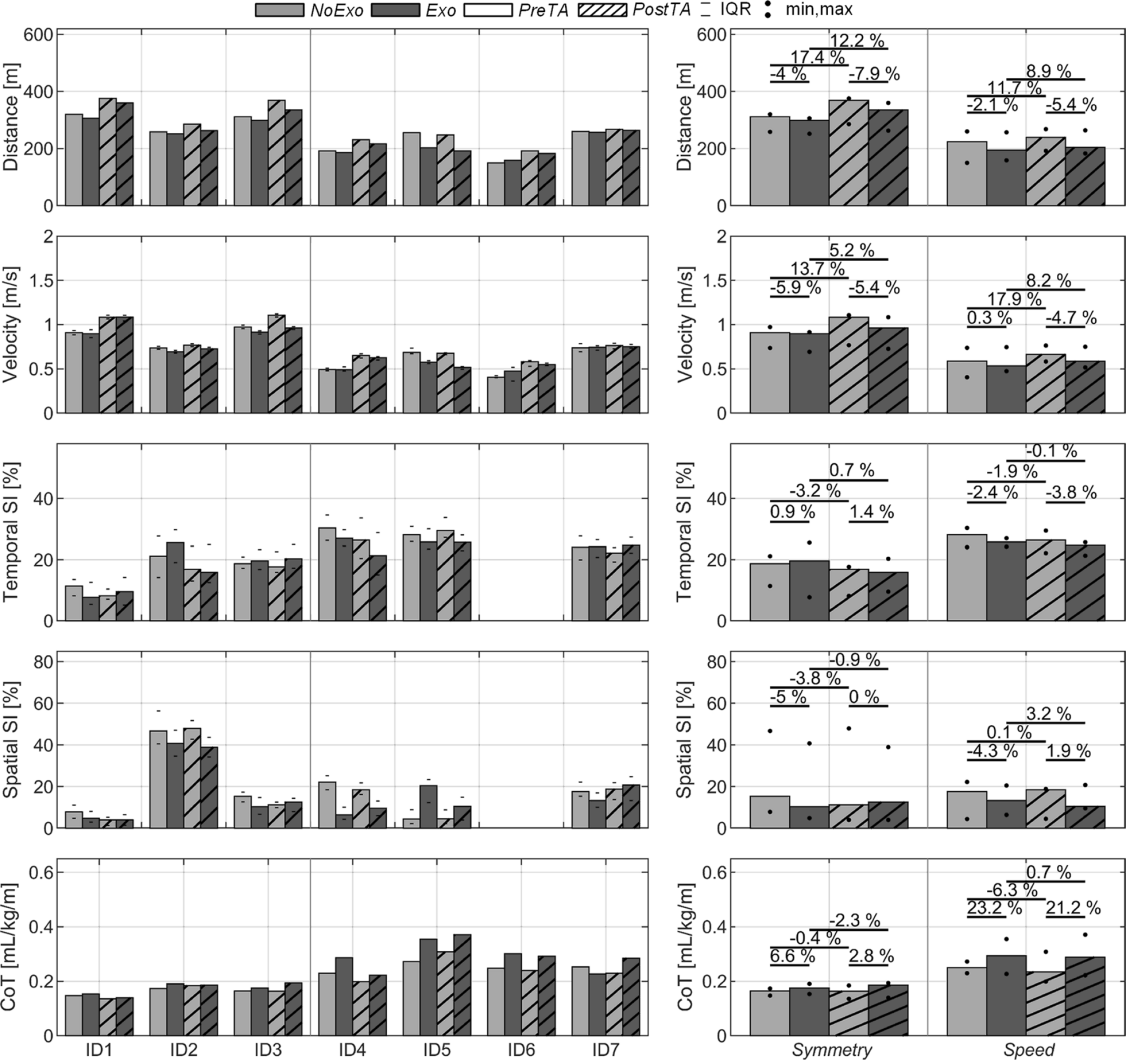
Barplots of the most relevant measurements obtained during the 6mWT. Data are reported individually on the left as median (IQR) and grouped on the right as median (min, max). All results are shown for *NoExo and Exo* conditions during *PreTA* and *PostTA*. The differences between the medians of each condition are shown in %

The most clinically relevant results regarding distance, walking velocity, symmetry, and CoT are summarized in Fig. 3, which reports individual and group results for each 6mWT (namely *PreTA* and *PostTA* in both *Exo* and *NoExo* conditions). Moreover, Table 2 reports additional information on cadence, stride length, Borg, and HR. The spatiotemporal results of ID6 are missing because the use of crutches was not compatible with the Optogait system.

**Table 2:** Additional performance results during the 6mWT

		Cadence [steps/min]		Stride length [cm]		CR10-Borg scale		HR [min^−1^]		%HRmax [%]	
		NoExo	Exo	NoExo	Exo	NoExo	Exo	NoExo	Exo	NoExo	Exo
ID1	*PreTA*	84.6 (82.8–86.3)	85.1 (82.8–86.3)	128 (125–132)	126 (123–130)	2	3	84.4	90.3	56.6	60.6
	*PostTA*	89.2 (88.0–90.2)	92.0 (90.0–93.3)	147 (145–149)	142 (138–144)	7	6	86.5	93.1	58.0	62.5
ID2	*PreTA*	84.3 (82.1–87.0)	82.0 (77.8–84.7)	105 (101–108)	103 (100–105)	5	5	136.2	136.4	85.7	85.8
	*PostTA*	82.1 (80.6–83.3)	78.7 7.0–81.2)	114 (111–116.8)	112 (109–113)	3	3	136.6	133.2	85.9	83.8
ID3	*PreTA*	91.5 (90.0–92.6)	89.9 (88.0–91.3)	127 (126–129)	121 (118–123)	1	2	106.0	113.2	63.1	67.4
	*PostTA*	95.4 (94.5–96.2)	91.5 (89.4–93.0)	139 (136–140)	125 (123–127)	3	3	114.4	119.0	68.1	70.8
ID4	*PreTA*	68.2 (66.8–69.6)	69.9 (68.1–71.4)	95 (91–98.5)	88 (82–93)	3	4	101.3	101.4	58.2	58.3
	*PostTA*	73.5 (71.4–74.9)	74.4 (72.4–76.4)	105 (103–107.3)	98 (96–102)	3	4	110.5	104.0	63.5	59.8
ID5	*PreTA*	91.2 (88.9–93.6)	86.0 (84.1–87.9)	92 (90–95)	79 (77–81)	3	3	104.4	111.4	66.1	70.5
	*PostTA*	92.0 (90.0–94.5)	82.3 (80.8–84.1)	89 (87–91)	75 (71.5–77)	1	1	108.6	107.3	68.7	67.9
ID6	*PreTA*	–	–	–	–	2	2	106.2	111.0	73.8	77.1
	*PostTA*	–	–	–	–	3	1	100.8	106.6	70.0	74.1
ID7	*PreTA*	83.5 (80.54–88.17)	83.6 (81.5–86.2)	104 (102–107)	105 (104–107)	2	3	106.5	118.4	67.0	74.5
	*PostTA*	84.5 (83.5–86.0)	82.6 (81.3–84.1)	108 (107–110)	103 (100.5–105)	3	5	106.4	113.9	66.9	71.6
*Symmetry group*	*PreTA*	84.6 (84.3–91.5)	85.1 (82.0–89.9)	127 (105–128)	121 (103–126)	2 (1–5)	3 (2–5)	105.6 (84.4–136.2)	113.2 (90.3–136.4)	63.1 (56.6–85.7)	67.4 (60.6–85.8)
	*PostTA*	89.2 (82.1–95.4)	91.5 (78.7–92.0)	139 (114–147)	125 (112–142)	3 (3–7)	3 (3–6)	114.4 (86.5–136.6)	119 (93.1–133.2)	68.1 (58.0–85.9)	70.8 (62.5–83.8)
*Speed group*	*PreTA*	83.5 (68.2–91.2)	83.6 (69.9–86.0)	95 (92–104)	88 (79–105)	2.5 (2–3)	3 (2–4)	105.3 (101.3–106.5)	111.2 (101.4–118.4-)	66.5 (58.2–73.8)	72.5 (58.3–77.1)
	*PostTA*	84.5 (73.5–92.0)	82.3 (74.4–82.6)	105 (89–108)	98 (75–103)	3 (1–3)	2.5 (1–5)	107.5 (100.8–110.5)	107 (194–113.9)	67.8 (63.5–70.0)	69.8 (59.8–74.1)
All participants	*PreTA*	84.5 (68.2–91.5)	84.3 (69.9–89.9)	104.5 (92–128)	104.0 (79–126)	2 (1–5)	3 (2–5)	106 (84.4–136.2)	111.4 (90.3–136.4)	66.1 (56.6–85.7)	70.5 (58.3–85.8)
	*PostTA*	86.8 (73.5–95.4)	82.4 (74.4–92.0)	111.0 (89–147)	107.3 (75–142)	3 (1–7)	3 (1–6)	108.6 (86.5–136.6)	107.3 (93.1–133.3)	68.1 (58.0–85.9)	70.8 (59.8–83.8)

Results are shown for Cadence and Stride Length as median (IQR); for the Borg scale as the reported value; for Heart Rate (HR) as the steady-state value (mean of the last 3-min of the 6mWT); and for the percentage of Maximal Heart Rate (%HRmax) as the computed value as in (60).Cadence and stride length could not be computed for ID6 due to the crutches. The last three rows present the median (min, max) of each group (*Symmetry* and *Speed*) and of All participants

### Post-training effects (No Exo: PostTA vs. PreTA)

From *PreTA* to *PostTA*, both groups increased their distance walked and walking velocity by more than 10%. For the *Symmetry* group*,* distance improved by a median 17.4% (10.4, 18.5) and velocity by 13.7% (4.3, 19.0), with a concurrent cadence and stride length increase of 4.3% (− 2.5, 5.3) and 9.5% (8.6, 14.8), respectively. After the training, the *Speed* group increased their distance by 11.7% (− 3.1, 28.0) and their speed by 17.9% (− 1.4, 43.6). The *Speed* group presented a higher variability: for instance, ID4 and ID6, who received the complete training program, improved walking speed by 32.2% and 43.6% respectively whereas ID5 and ID7, who trained for half of the sessions, only presented small changes in velocity (− 1.4% for ID5 and 3.5% for ID7). On average, the median velocity of the *Symmetry* group was 54% higher than the *Speed* group, consistent with the different velocity-related instructions given to each group during the 6mWT: maximal speed for the former and self-selected speed for the latter.

Following training, the spatial and temporal SI respectively improved by 2.9% (0.9, 3.6) and 3.6% (− 0.8, 4.8) for the *Symmetry* group. The *Speed* group showed smaller SI improvements, with a median change of 1.6% (− 1.0, 3.0) for temporal SI and 0.1% (− 1.0, 3.1) for spatial SI, with higher across-participant variability: ID4, the only reported participant from the *Speed* group with a complete training program, increased both the temporal and spatial SI by more than 3% whereas ID5 and ID7 showed little or no improvements (− 1.0% and 1.5% for temporal SI and 0.1% and − 1.2% for spatial SI, respectively).

CoT at *PostTA* did not change significantly for the *Symmetry* group with respect to *PreTA*, as the difference was -0.4% (− 7.9, 6.4) whereas the *Speed* group presented a 6.2% (− 13.4, 13.12) lower CoT at *PostTA*. The high variability was due to ID5, the only subject who increased CoT at *PostTA* vs. *PreTA*, by 13.2%. Regardless of the test speed, the 6mWTs were sustained with HRs lower than 75% of the HR_max_ by all participants except for ID2 and ID7, who exhibited 85% and 77% of HR_max_ (respectively) at *PostTA in NoExo condition*.

### Effect of the exoskeleton (Exo vs. NoExo)

Walking velocity was reduced when wearing the APO (*Exo*): in the *PostTA* session, the median velocity reduction was 5.4% (− 13.0, 0.1) for the *Symmetry* group and 4.7% (-23.6, -1.8) for the *Speed* group compared to the *NoExo* condition. However, it was noteworthy that during the *PreTA* session, one participant of the *Speed* group walked approximately 17% faster in the *Exo* condition.

The effect of the exoskeleton (*Exo* vs. *NoExo*) on symmetry was highly variable both across and within groups at *PostTA*: for the temporal SI, the median difference was 1.4% (− 1.0, 2.7) for the *Symmetry* group and − 3.8% (− 5.1, 2.6) for the *Speed* group. For spatial SI, the median difference was 0% (− 9.0, 1.3) for the *Symmetry* group and 1.9% (− 8.9, 6.0) for the *Speed* group.

Finally, the CoT increased while wearing the APO in all participants compared to the *NoExo* condition, except for ID7 at *PreTA*. At *PreTA*, the *Exo* CoT increased by 6.6% (4.1, 10.1) relative to *NoExo* for the *Symmetry* group and for the *Speed* group by more than 20% (-10.3, 30.2). Moreover, at *PostTA* the *Exo* vs*. NoExo* CoT difference was essentially unchanged from the *PreTA* session: 2.8% (1.0, 18.5) for the *Symmetry* group and more than 20% (11.8, 24.1) for the *Speed* group. The results on the CoT did not always match subjective evaluations obtained from the CR10-Borg scale (Table 2): contrary to the CoT data at *PostTA*, ID2 and ID4 reported less fatigue after performing the 6mWT in the *Exo* compared to the *NoExo* condition.

### Gait analysis (GA)

**Fig. 4:**
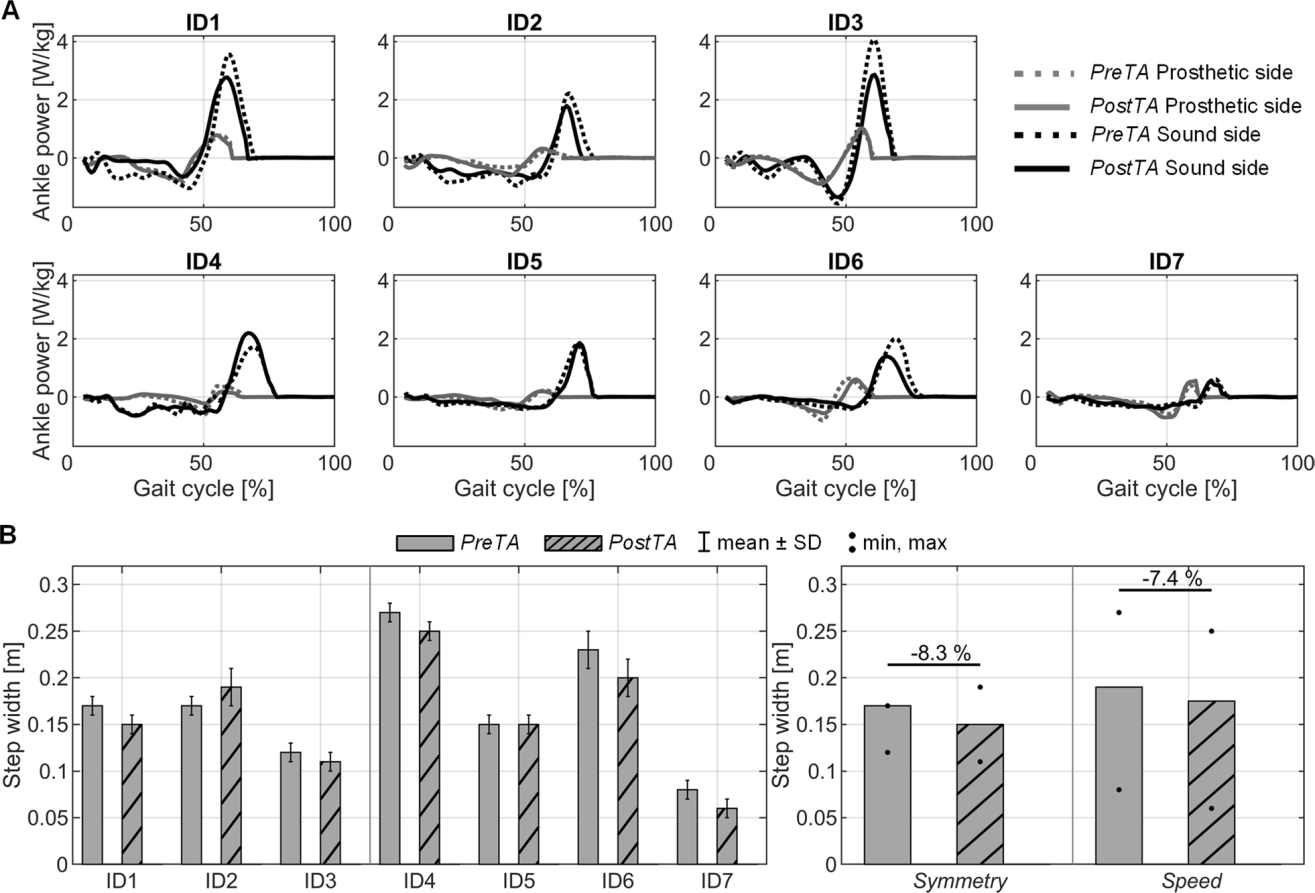
Relevant results during the Gait Analysis (GA). **A.** Ankle power for each participant as a function of the gait phase on the prosthetic and sound sides. Dotted and solid lines represent respectively data from *PreTA* and *PostTA*. **B.** Barplot of the step width. Data are reported individually on the left as mean ± SD and grouped on the right as median, (min, max). All results are shown for *PreTA and PostTA* in *NoExo*. The differences between the medians of each condition are shown in %

The most evident results of the GA were observed in the ankle power during the push-off phase. As shown in Fig. 4, participants in the *Symmetry* group (ID1-3) were able to reduce the maximum ankle power produced by the sound limb after the training by 22% (19.3, 29.6), while maintaining or increasing the walking velocity. A similar result was obtained for ID6, with a power decrease of 30.8% at *PostTA* compared to *PreTA*, but not for the other members of the *Speed* group.

Step width was also affected by training with the APO, decreasing by 8.3% (− 11.8, 11.8) for the *Symmetry* group and by 7.4% (0.0, 25.0) for the *Speed* group. Furthermore, 5 participants (ID1, ID3, ID4, ID6, ID7) decreased their step width by 11.8% (7.4, 25.0). One participant (ID5) did not show significant changes in step width, while the only participant who increased the step width (11.8%, ID2) had major improvements in terms of RoM and temporal symmetry, increasing the RoM on the prosthetic side by 14% while improving temporal symmetry by 3%.

## Discussion

This study investigated as main outcomes (1) the feasibility of a one-month mobility-based overground gait training program with the APO and (2) the effects of the APO-assisted training program on gait speed and symmetry in community-ambulating TFAs. Secondary outcomes included the effects of the training (*PostTA* vs. *PreTA*) on other gait parameters (metabolic cost, step width, and gait kinematics) and modifications in these parameters when walking with the APO compared to walking without it (*Exo* vs. *NoExo*). Indirectly, this study also verified the safety of the training protocol and the reliability of the APO.

First of all, the APO was shown to be safe and reliable when used throughout the 10-month clinical study with TFAs. The only hardware failure detected was the breakage of the plastic shell of the spine brace. The team of APO designers hypothesized that the failure was the consequence of an inappropriate way of manipulating the robot during the don-/doffing phases. After a careful analysis, experimenters were advised to grasp the APO through the carbon-fiber frame instead of the spine brace, to avoid overloading the shell. Importantly, this failure occurred progressively with a slow degradation of the efficacy of the robot-to-human energy transfer and never affected users’ safety.

Participants did not mention any discomfort or injuries specifically related to the robot during or after the training program. One participant presented superficial skin lesions at the start of the study. After the initial evaluation, experimenters judged that this issue did not affect the mobility of the participant and thus did not hinder participation in the training program. In that case, residual limb skin health was monitored throughout the study, and the participant was allowed to train with more frequent rests if needed. Importantly, no participant missed a training session due to residual limb complications. Moreover, skin issues are common on residual limbs of transfemoral amputees and are not believed to be specifically related to training [61].

A primary result of this study was that at the end of training *(PostTA vs. PreTA)*, participants walked longer distances at a considerably faster speed while not wearing the exoskeleton (*NoExo).* Velocity was evaluated in the middle of the corridor, thus the differences between improvements in total distance walked vs. steady-state speed (respectively 17% vs. 14% for the *Symmetry* group and 12% vs. 18% for the *Speed* group) may be related to the time spent to turn around at the end of each corridor, which could have been more challenging for the *Speed* group participants due to their overall lower mobility and different prosthetic components.

Gait speed is the simplest and one of the most-reported metrics of gait performance in previous studies related to the assessment of specific exercise programs for lower-limb amputees [27, 62]. Also, gait speed plays a fundamental role in successfully engaging TFAs in outdoor everyday life and recreational activities, representing the most relevant self-perceived objective for amputees enrolling in a rehabilitation program [63, 64]. Even though the uncontrolled nature of the study makes it impossible to isolate the specific contribution of the APO assistance in achieving this result, this study represents the first proof of feasibility that a low-to-moderate-intensity, relatively-short-term robotically assisted rehabilitation program can result in functionally-significant gait speed improvements for lower-limb amputees in overground walking. The fact that improved gait speed (maximal and self-selected) was obtained independently from the initial walking ability of the participants and from the assistive strategy further encourages future studies aiming at exploring the potential of this technology.

Besides speed, training with the APO improved the gait pattern. At the end of the training (*PostTA* vs. *PreTA*), all participants who completed the training protocol presented more symmetrical walking patterns, regardless of which APO assistive strategy was used, which was consistent with literature, as training programs have been shown to reduce the gait deviations of amputees [21, 27]. The amount of time necessary to obtain such results with the APO is yet unknown but could be investigated by measuring symmetry and speed during all training sessions. Similarly, 5 out of 7 participants in both groups decreased step width, which may be a consequence of having recovered a more stable and confident gait pattern and/or the constraining effect induced by the tight attachment of the APO linkages to the participants’ thighs. Indeed, despite the APO’s passive ab/adduction DoF, the device still partially constraints hip motion in the frontal plane. Interestingly, the reduction of the ankle power on the sound side observed for all three participants of the *Symmetry* group and only one of the *Speed* group is indicative of a trend leading to a more physiological ankle power profile, as TFAs usually compensate the lack of power on the prosthetic side by increasing the ankle power on the sound side during the push-off phase [5]. These biomechanical improvements were larger in the *Symmetry* group, hence it is hypothesized that the combination of (1) the assistance targeting TFAs’ abnormal stance durations, (2) the participants’ higher mobility, (3) the prosthesis type (ESAR or rigid foot), and (4) their previous familiarization with the device conferred a more effective gait than for participants with lower mobility levels [65]. These results thus encourage a further investigation of the underlying determinants of the effects of robotic assistance on gait kinematics and dynamics.

The rehabilitative results obtained in this study are difficult to compare to existing literature since training programs are quite variable in terms of target patients, training modality, volume, and intensity [21, 62]. In fact, the closest studies to our clinical intervention are treadmill-based gait training programs to train community-ambulating TFAs to restore walking abilities [26, 27]. The results of [27] showed rehabilitative effects similar to ours after 4 weeks of intense home-based treadmill training (speed 55% higher than self-selected). The study found: (1) maximal and self-selected speeds increased by 11% and 13%, respectively, (2) temporal symmetry improved by 1.5% only at self-selected speed, (3) spatial symmetry improved by 6% at self-selected speed and 0.9% at maximal speed. Moreover, to the authors’ knowledge, several studies have analyzed kinematics of TFAs after special gait re-education [29, 66], but the only biomechanical analysis comparable to our study was presented in [26], where a single participant was trained for three weeks with VR. The participant showed improved kinematics in the frontal plane in trunk and pelvis motion and hip ab/adduction [26], which were not seen in the current study.

Hip exoskeletons presented in the literature have mostly been directed at the elderly, post-stroke subjects or other neurologically-impaired individuals [38, 40, 41], but recent literature has reported the results of training 2 TFAs (one of them bilateral: trans-tibial on the left side and trans-femoral on the right side) for 5 days including 20 min of training with a powered hip orthosis combined with 40–100 min of conventional rehabilitation [67]. Both subjects improved self-selected speed, step length, cadence and hip RoM, although the small number of subjects, absence of control group, and the balance of 60–80% conventional rehabilitation in the intervention makes it hard to isolate the effects of the exoskeleton. Indeed, our study has similar limitations, with the main limitation of this study being the absence of a control group performing an equivalent training program without the exoskeleton. As the participants informally reported to have walked more than usual due to the training period, the observed beneficial effects might also be related to the increased exercise volumes. Another important consideration concerns the major across-participant variability in several of the reported outcomes. Though the experimental setup was almost identical for all subjects to reduce the number of confounding factors, the protocol was conceived to deliver subject-dependent assistive profiles based on the subject’s mobility, and sometimes the participants’ characteristics and needs required individual adjustments and minor deviations from the protocol, as in the case of ID5 an ID7, who completed half of the training sessions with a simplified assistive profile. Nevertheless, it was judged by the clinical study team that the current intra-group variability in overall health or prosthetic device was reflective of the inherent clinical heterogeneity of the TFA population and that differences in overall participant mobility between the two groups was large enough to justify different training outcomes. Also, since all participants were community ambulators, it is possible that a pool of participants with lower mobility might have required different assistive profiles or benefitted differently from the APO. Finally, all subjects dedicated less than 20% of the training session to secondary activities (stairs and sit-to-stand transitions), and though it is inferred that their influence on the results is minimal, it is not possible to isolate their precise contribution. All these considerations, combined with the relatively small number of volunteers and their split into two subgroups, constitute a significant limitation to the statistical power of the achieved results.

Concerning the aerobic capacity, results were highly variable across participants: the CoT difference between *PostTA* and *PreTA* in *NoExo* ranged from − 13.4% to + 13.5%. Of note, aerobically intensive training was not one of the objectives of this study, and the walking speed and HR were monitored by the experimenters during the training sessions to maintain a low-to-moderate intensity, which usually requires longer training to obtain significant improvements in aerobic capacity [68]. A loading effect was observed when wearing the APO, as most subjects presented a higher metabolic cost and a slower walking speed in *Exo* compared to *NoExo* condition. Only one subject walked significantly faster when wearing the exoskeleton (speed increased by 0.07 m/s), and only in the *PreTA* session. As the instructions to complete the 6mWT were identical in the two assessment sessions, this unexpected result might have been related to a psychological bias of the participant on the first day of assessment. Indeed, this participant was enthusiastic about the device and though the CoT increased by more than 20% when wearing the device at *PreTA*, the perceived effort assessed by the CR10-Borg scale was identical between *NoExo* and *Exo* conditions*.* Also, similarly to what has been reported in literature [51], generally the CoT was not consistent with the subjective results of CR10-Borg scale: at *PostTA*, two subjects reported less fatigue even though their CoT increased by 2.8% and 22.0% when walking with the APO.

The slower walking speed when wearing the APO might be related to the order of the 6mWT (the *Exo* condition was always performed after the *NoExo* condition), or to the additional weight carried on the back that increases the inertia of the walking system and demands a higher braking and accelerating force [69–71]. Additionally, in order to perform the 6mWT, participants were requested to decelerate and accelerate at the end of each corridor, which can be considered a pseudo-oscillating walking speed, previously shown to increase metabolic cost [72]. Thus, the experimental setup of the 6mWT might have been detrimental for assessing gait efficiency with the APO. In fact, when using different versions of the device in a more structured environment such as walking on a treadmill, elderly subjects and TFAs were able to reduce the metabolic cost when being assisted by the device [41, 73]. The different results achieved with this version of the APO may also be related to the device itself, as the particular position of the Center of Mass or the encumbrance of this version might have negatively affected the inertia of the human–robot system. It could also be possible that the APO was not unloaded properly at the hip level or that the power transfer was not optimal due to the performance of the adopted spine brace and/or the timing of the assistive torque profiles [74, 75].

For further consideration, the *Exo* vs. *NoExo* differences in the metabolic cost were highly variable across subjects and did not seem to improve between the start and the end of the training. This contrasts with literature findings showing that for healthy subjects a familiarization of two or three sessions is enough to optimize metabolic cost and reach a steady difference between with and without the exoskeleton [76, 77]. Our different result might be partly due to *PreTA* being the second session of practice with the exoskeleton, as participants had already walked with the exoskeleton during the Tuning session. It would thus have been interesting to measure metabolic cost also before the *PreTA* to quantify the familiarization effects of that first session. Moreover, the prolonged training period may have had effects on the aerobic training level of the participants, thus mixing the effects of exoskeleton familiarization with effects of aerobic training. Finally, the assistance strategy was not designed to target improvements in metabolic cost; rather, the tuning procedure was mostly based on kinematic considerations, whereas in [73] the authors used a tuning strategy that verified in real-time the effect of assistance on the participant’s energetic CoT. Overall, the results of this study on the metabolic cost seem to indicate that different training protocols and dedicated assistive strategies might be necessary to improve aerobic fitness and gait efficiency of TFAs. In our study, the tuning session required that subjects walked for a median time of 40 min with the exoskeleton, so in order to incorporate additional considerations of metabolic cost while also including participants with lower mobility in the protocol, the tuning procedure might need to be optimized and automated [78].

To overcome these limitations, future studies should include a control condition, a prolonged training period, and a larger sample size to quantify the effect of training with the orthosis and ensure a clearer interpretation of results by minimizing the effects of inevitable inter-participant variability. Testing patients with alternative disorders, such as post-stroke individuals may also be interesting since it is expected that participants with different lower-limb impairments will adapt differently to the assistance of the APO. Moreover, it might be necessary to record additional physiological parameters such as muscle activation, as well as the comparison between the walking abilities during the *Exo* condition in AM compared to TM. Additional outcome measures could help to better understand and localize the specific benefits that each participant obtains from the device and to fine-tune the personalized assistance or modify the participant instructions accordingly. Since the tuning of the assistance seems critical for improving metabolic efficiency during walking, dedicated studies will be carried out in the future paying special attention to applying human-in-the-loop optimization to find the most suitable set of tuning parameters. Finally, kinematics and gait efficiency should be investigated in more controlled conditions such as on a treadmill, which is the most common setup found in the literature for metabolic assessment with TFAs and other lower-limb disorders [27, 39, 40].

## Conclusions

This pilot study investigated the feasibility of applying a wearable robotic orthosis (APO) to assist TFAs in an overground gait rehabilitation program and provided meaningful information on which gait parameters could be prioritized as rehabilitative outcomes to be pursued by future APO-assisted programs. Results showed that tailored assistance could affect both gait speed and symmetry, which could be considered as primary goals when training TFAs with the APO. However, wearing the APO while training decreased walking speed and increased energetic demand relative to walking without it. Future studies will therefore integrate a lighter and less encumbering version of the APO and optimize tuning procedures with specific assistive strategies dedicated to reducing metabolic cost. An important limitation of this study is that it did not directly compare the effectiveness APO training with respect to traditional overground walking and subsequent studies should aim to compare a similar APO-mediated training program with a control condition in a randomized trial.

## Data Availability

The datasets used and/or analysed during the current study are available from the corresponding author on reasonable request.

## Abbreviations

TFA: Transfemoral amputee
ADLs: Activities of daily-living
APO: Active pelvis orthosis
ID1-8: Identification number of subjects
RoM: Range of movement
FPGA: Field-programmable gate array
TM: Transparent mode
AM: Assistive mode
ESAR: Energy storing and return prosthetic foot
*Mech*: Mechanical knee joint
*Electr*: Electronic knee joint
GA: Gait analysis
*PreTA*: Pre-training assessment
*PostTA*: Post-training assessment
6mWT: Six-minute walking test
*NoExo*: Condition in which the subject does not wear the exoskeleton
*Exo*: Condition in which the subject wears the exoskeleton
SI: Symmetry index
VO2: Volume of oxygen uptake
CoT: Energy cost of transport
RER: Respiratory exchange ratio
BM: Body mass
HR: Heart rate
HRmax: Maximal heart rate
